# Evaluation of permeability applicability based on continuum mechanics law in fluid flow through graphene membrane

**DOI:** 10.1038/s41598-019-49131-y

**Published:** 2019-09-03

**Authors:** Taro Yamada, Ryosuke Matsuzaki

**Affiliations:** Tokyo University of Science, 2641 Yamazaki, Noda, Chiba, 278-8510 Japan

**Keywords:** Mechanical engineering, Graphene, Nanoscale materials

## Abstract

Graphene is expected to be used in separation applications such as desalination. However, it is difficult to predict the flow phenomena at the nanoscale using the conventional continuum law. Particularly at a Knudsen number (*Kn*) of >0.1, which is applied in filtration, it has been reported that not even slip boundary conditions can be applied. In this study, to identify the parameters that affect the applicability of the continuum law, we conducted a fluid permeation simulation using graphene. The deviation of the permeability from that of the continuum model was calculated by changing the channel width, fluid temperature, and fluid type. The result showed that the channel width has the largest influence among the three factors, and that the magnitude of the divergence is sorted out based on the Knudsen number. Therefore, the permeability can be predicted even at the nanoscale where the continuum law cannot be applied.

## Introduction

Focusing on an interaction with a fluid, graphene has very low friction on its surface, and some researchers have used graphene to generate nanoscale films, such as mechanical pressure sensors^1^ and cell compartments^2^. Particularly in the case of a liquid, a high permeability has been reported, and it is being investigated whether a liquid can be used for atomic-level filtration such as desalination^3^ or ion separation^4,5^. For example, Geim *et al*.^6^ evaluated the permeability of graphene membranes prepared experimentally with water and other fluids. Although it does not permeate a fluid such as He, a permeability of 10^10^ times larger than in the case of water has been reported. However, because a flow at the nanoscale has a relatively large influence on the wall surface, it is empirically known that such a flow deviates from the basic continuum law, including a continuous equation or non-slip condition^7^. Therefore, even in the flow in a graphene filtration membrane, these basic laws cannot be applied, and there is a possibility that the desired permeation level cannot be obtained.

To understand the flow phenomenon at the nanoscale, a large number of studies clarifying the flow mechanism both experimentally and analytically have been carried out in recent years. In the case of liquid permeation, it is known that a liquid deviates from the flow at the macro-scale as compared with a gas because molecules are always adsorbed on the wall surface in the case of a liquid, and a molecular effect such as a slip on the wall surface becomes more significant. It is known that such deviations begin to occur when the Knudsen number (dimensionless number divided by the liquid’s molecular diameter based on the characteristic length of the flow path) exceeds 10^−3^ ^8,9^. Particularly within the range of 10^−3^–10^−1^, it is empirically known that the continuum model can be applied by providing the wall slip boundary conditions^7,10–13^. For example, Bhatia *et al*.^14^ developed a model incorporating transducers to calculate the permeability owing to a diffusion of the porous graphene membranes. This model is particularly accurate for low-density fluids, and there is an advantage in that the influence of a plurality of holes can be superimposed. However, to obtain the boundary conditions on the wall, it is necessary to introduce a complex function representing the thermodynamic state of the fluid and wall. Therefore, as another approach, a model that calculates the slip length using the Green-Kubo relationship has been proposed^15^. The slip length is calculated as the ratio of the shear viscosity to the friction coefficient, which can be calculated from an MD simulation in a state of equilibrium. Because the slip length obtained can be used as a Navier slip boundary condition, the handling of a flow close to the macro scale is possible^16^. However, it has been reported that the slip boundary conditions cannot be applied when the pores are smaller than five molecules, that is, *Kn* = 0.2^17^.

In this study, we identify those parameters that affect the applicability of the continuum law in a nanoscale flow. In addition, the fluid permeability is verified assuming filtration and resin impregnation applications. Specific parameters that can be adjusted at the time of the flow path is designed are focused upon, and the flow path width, fluid temperature, and type of fluid molecules are changed. Furthermore, we propose a method for predicting the magnitude of divergence using the affected parameters.

## Results

### Hydrodynamic model

In a nanoscale flow, the Knudsen number (*Kn*) is used to classify the flow mechanism, as shown in Table 1 ^18^. Here, *Kn* is defined as the ratio between the mean free path *λ* of a fluid molecule and the representative length *L*_*s*_.

1 $$Kn=\frac{\delta }{{L}_{s}}$$

**Table 1:** Knudsen number regimes.

*Kn*	Flow condition	Continuum mechanics
*Kn* < 10^−3^	Continuum flow	Applicable
10^−3^ < *Kn* < 10^−1^	Slip flow	Applicable
10^−1^ < *Kn* < 10	Transition regime	Not applicable
10 < *Kn*	Free-molecular flow	Not applicable

For a liquid, because the distance between fluid molecules is smaller than that of gas molecules, lattice spacing *δ* is used instead of the mean free path^18^. The value of *δ* is generally used as Lennard-Jones diameter or Van der Waals diameter^19^. This value can be applied when the molecular shape is close to a sphere, similar to a monoatomic molecule, but cannot be applied to a polymeric fluid such as resin because multiple Van der Waals diameters of the molecules may occur (shortest and longest sides). Therefore, we approximate the volume occupied by a single molecule using a sphere and apply the equivalent molecular diameter calculated.

2 $${d}_{e}={(\frac{6M}{\pi {N}_{A}\rho })}^{1/3}$$

where *M* is the molecular weight of the fluid molecule, *N*_*A*_ is an Avogadro constant, and *ρ* is the density.

**Figure 1:**
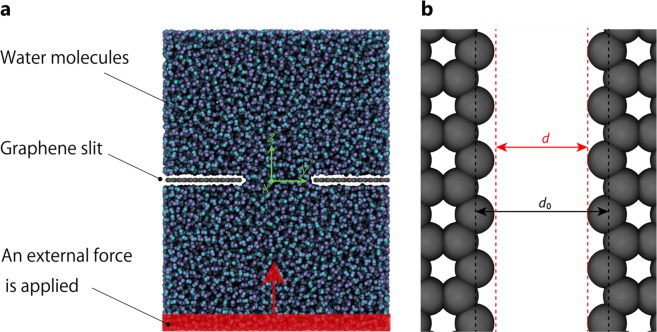
Water molecules permeating between graphene slits. (**a**) Schematic of computational setup. The gray, cyan, and blue colors represent the carbon, oxygen, and hydrogen atoms, respectively. The graphene slit is located at the center of the unit cell. The red shading represents the region where the external force is applied. (**b**) Definition of slit width *d*.

We consider a single-layer graphene slit model with width *d*, as shown in Fig. 1(a). For *Kn*, the slit width *d* (0–5.0 nm) is used for the representative length *L*_*s*_. However, because a strong repulsive force acts on the fluid molecules at the end of the slit, *d* differs from distance *d*_0_ between the carbon atoms. Therefore, as shown in Fig. 1(b), *d* is defined as the distance *d*_0_ excluding the distance *d*_*h*_ where no molecules exist.

3 $$d={d}_{0}-{d}_{h}$$

There are two types of liquid molecules applied, water and ethanol. Their molecular diameters *d*_*e*_ are found to be approximately 0.38–0.58 nm based on a calculation described later, with a *Kn* of 0.12–5.0, which corresponds to the transition region shown in Table 1. In this region, when *d* is wide, the continuum law can be applied and the applicability narrows, and the flow can be expected to transition into a molecular flow.

The permeability is an index indicating the flow capability of a fluid, in which larger values allow permeation at lower energy. This is expressed based on the ratio of the flow rate *Q* permeating through the membrane and the difference in pressure Δ*P* before and after permeation.

4 $${K}_{MD}=\frac{Q}{{\rm{\Delta }}P}$$

When flowing between 2D micro slits of width *d* under non-slip conditions, permeability *K*_*eq*_ can be obtained as follows by applying a Stokes approximation to the Navier-Stokes equation from the viewpoint of continuum mechanics^20^.

5 $${K}_{eq}=\frac{\pi {d}^{2}}{32\,\mu }$$

where *μ* is the viscosity of the fluid, and *d* is the slit width. The density and viscosity change with a change in temperature, and thus we use the value in a bulk state obtained from the equilibrium MD simulation (see the Methods section) with only fluid molecules.

### MD results

**Figure 2:**
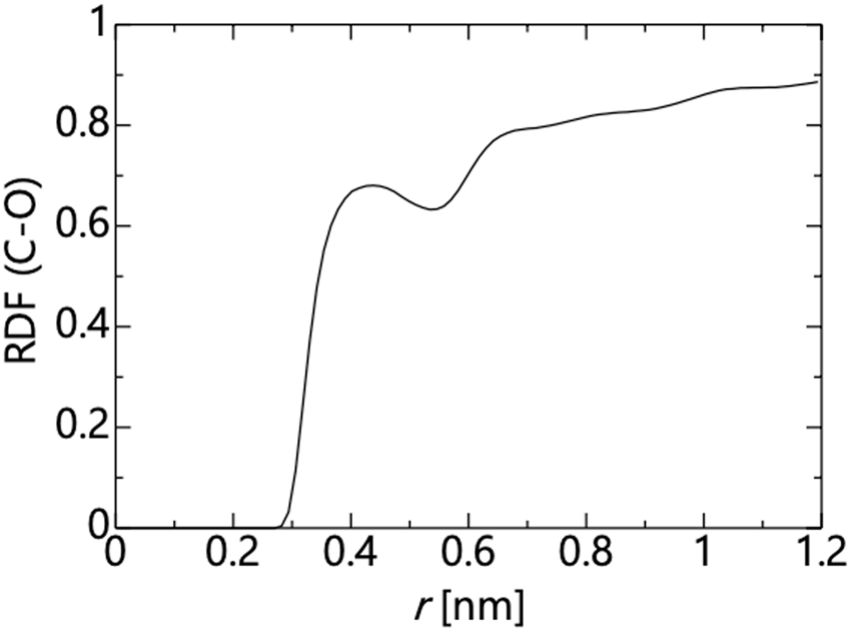
Radial distribution function of C(graphene)-O(H_2_O) at *d*_*0*_ = 3.0 nm. Similar results are obtained at other channel width.

First, we investigated the effects of the channel width on the applicability of the continuum law. We used the model shown in Fig. 1(a), with the temperature set to 300 K, the molecular type set to water, and the distance between carbon atoms set to 0.4 < *d*_0_ < 3.0 nm. Figure 2 shows the radial distribution function (RDF) of carbon atoms of graphene and oxygen atoms of water molecules. As a representative example, only the result at *d*_0_ = 3.0 nm is shown, whereas similar results are obtained at other values of *d*_0_. At a distance of approximately 0.25 nm, the value of RDF is zero. This indicates that graphene and water molecules cannot exist at a distance of less than 0.25 nm. Therefore, *d*_*h*_ = 0.5 nm in consideration of the elimination effect at both ends.

**Figure 3:**
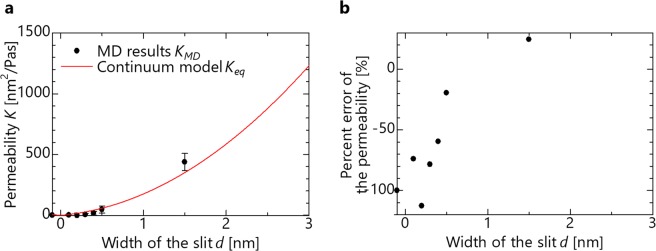
Effect of the channel width at 300 K with water molecular. (**a**) Permeability as a function of the channel width. (**b**) Percent error of permeability as a function of the channel width.

Second, the permeability obtained through the flow calculation are shown in Fig. 3(a) using the continuum model of Eq. (5). The permeability obtained through an MD calculation are lower than that of the continuum model at most channel widths, and is impermeable at *d* = −0.1 nm (*d*_0_ = 0.4 nm). Considering the reports that the viscosity increases from the bulk state owing to the change in the molecular structure between nanochannels^14,15^, *K*_*eq*_ decreases from Eq. (5), and this result is therefore considered to be due to the use of the bulk viscosity. To obtain the tendency regarding the degree of divergence, the percent error between the permeability of the MD and that of the continuum model equation is shown in Fig. 3(b). The permeability obtained from the MD calculation in Eq. (4) is compared with that obtained from the continuum model in Eq. (5). Our study is based on the assumption that the MD calculation value is the actual value. That is because the continuum model has been reported to deviate from the actual values, but it has been reported that the MD calculation can reproduce actual values with careful setup. This percent error expresses how much the MD result deviates from the continuum model equation and can be calculated by the following equation.

6 $${\rm{Percent}}\,{\rm{error}}\,{\rm{of}}\,{\rm{the}}\,{\rm{permeability}}=\frac{{K}_{MD}-{K}_{eq}}{{K}_{eq}}$$

When the value is close to 0, the continuum model can be applied. When the value is positive, the MD result is larger, and when it is negative, the MD result is smaller than that obtained using the continuum model. The percent error is −10% or more when *d* > 1.5 nm when the channel is wide, but is less than −50% at d < 0.5 nm as the channel narrows. In other words, the divergence increases as the channel width decreases. The reason why the divergence increases is described later in the topic of molecular orientation research.

**Figure 4:**
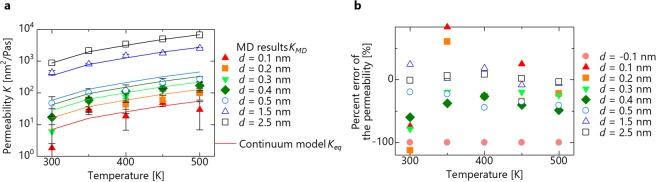
Effect of the temperature with water molecular. (**a**) Water permeability as a function of temperature. Each line indicates a theoretical equation. (**b**) Percent error of water permeability as a function of temperature.

Next, we investigate the influence of the fluid temperature on the applicability of the continuum law. The temperature is 300 < *T* < 500 K, the molecular type is water molecules, and the distance between carbon atoms is 0.4 < *d*_0_ < 3.0 nm. The relationship between temperature and permeability is shown in Fig. 4(a). Because *d* = −1.0 nm is impermeable under all conditions, they are not plotted. The permeability increases as the temperature increases. This is consistent with the trend in the continuum model in which the decreasing viscosity with an increase in temperature increases the permeability. The percent error from the continuum model is shown in Fig. 4(b). At first sight, this graph shows no relationship between temperature and percent error. In order to show quantitatively that there is no relationship between temperature and percent error, we investigated whether each slope has an advantage in all channel widths by conducting a t-test. As a result, the null hypothesis that the slope is not 0 could not be rejected at *p* = 0.05 for all channel widths. Therefore, the percent error is not affected even if the temperature rises in the measured temperature range.

**Figure 5:**
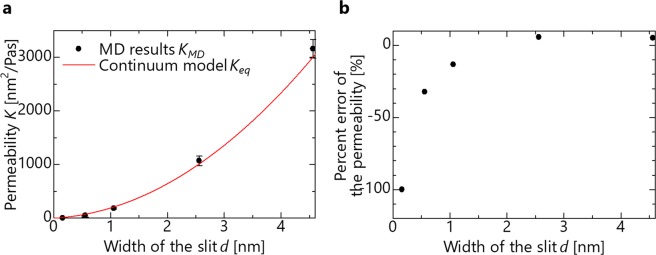
Effect of the channel width at 300 K with ethanol molecular. (**a**) Ethanol permeability as a function of the channel width. (**b**) Percent error of ethanol permeability as a function of the channel width.

Next, we investigated the influence of the type of fluid molecules on the applicability of the continuum law. The temperature is only 300 K because it was clarified in the previous section that the temperature does not affect the percent error of the permeability. The molecular type here is ethanol, and the distance between carbon atoms is 0.6 < *d*_0_ < 5.0 nm. Similar to water molecules, *d*_*h*_ = 0.44 nm is obtained from the RDF calculation. As shown in Fig. 5(a), the permeability obtained from the MD calculation at most channel widths is lower than that of the continuum model and is impermeable at *d* = −0.16 nm (*d*_0_ = 0.5 nm). This result shows the same tendency as the case of water molecules. Figure 5(b) shows the percent error from the continuum model. Similar to water molecules, the divergence increases as the flow channel width decreases. These results indicate that the applicability of the continuum law shows the same tendency even if the type of molecules changes.

**Figure 6:**
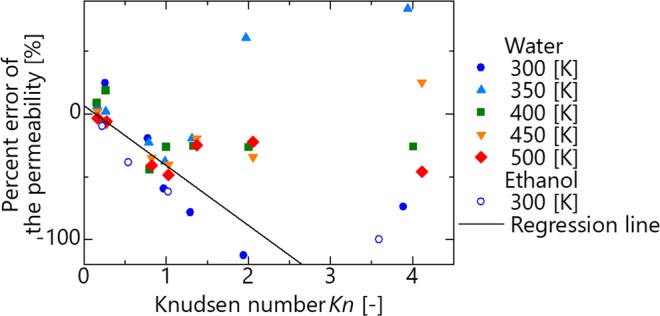
Percent error of the permeability as a function of the Knudsen number.

**Figure 7:**
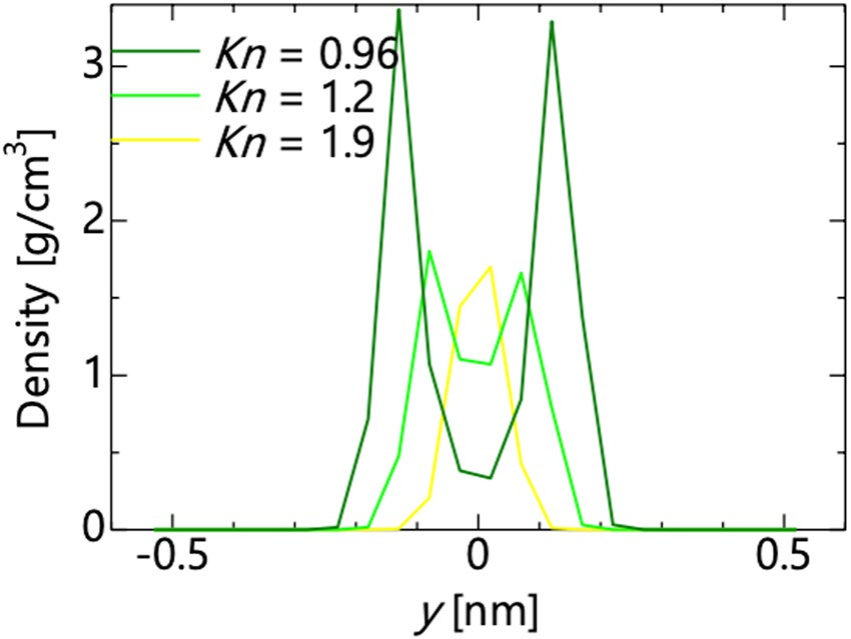
Density profile of water molecules at 300 K.

The percent error in the permeability described above is sorted based on the Knudsen number, as shown in Fig. 6. When *d* < 0, the molecules are impermeable under all conditions and are excluded. When *Kn* < 1.0, a negative correlation coefficient of less than −0.7 is confirmed based on the Knudsen number and percent error. Therefore, when *Kn* < 1.0, the applicability of the continuum law can be predicted as a linear expression of the Knudsen number. When *Kn* > 1.0, it can be confirmed that the percent error approaches −100% as *Kn* increases, although the variation is larger than *Kn* < 1.0. This suggests that the flow mechanism changes with *Kn* < 1.0 and *Kn* > 1.0. Figure 7 shows the density profile between slits at *Kn* = 0.96, 1.2, and 1.9 where the variations are suddenly changed. As the Knudsen number increases, the number of high-density peaks decreases from two to one. Consequently, the flow mechanism changes when changing from permeation in multiple layers to permeation in a single layer.

**Figure 8:**
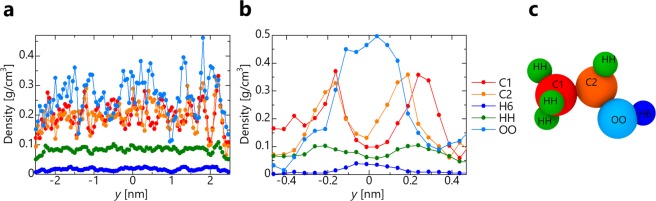
Density profile of each atom constituting ethanol molecule. (**a**) *d* = 5.0 nm (*K*_*n*_ ≈ 0.12). (**b**) *d* = 1.0 nm (*K*_*n*_ ≈ 1.03). (**c**) Molecular model of ethanol.

We investigated the influence of the channel width, temperature, and molecular type, and found that the divergence from the continuum model becomes large particularly as the channel width narrows. To investigate this cause, we focus on the molecular orientation during permeation. Figure 8 shows the density profile between slits for each atom in the ethanol molecules. In the case of water molecules, the number of constituent atoms is smaller than that of ethanol, and it is difficult to obtain the orientation. Thus, we focused on ethanol. Each color of the graph corresponds to the atoms in Fig. 8(c). In the case of *Kn* = 0.1, the distribution is close to uniform. However, in the case of *Kn* = 1, C1 and C2 have a high density at *y* = ±0.2 nm, and H6 and OO have a high density at *y* = 0 nm. This result indicates that the methyl group (-CH3) is more likely to adsorb the graphene than the hydroxyl group (-OH), and this adsorption layer has different characteristics from those of a bulk liquid, and the influence of the adsorption layer becomes remarkable as the Knudsen number increases. In other words, because the influence of the liquid layer in contact with the wall surface becomes relatively large, the proportion of adsorption layers having different molecular structures increases, as does the viscosity. Therefore, the MD result deviates from Eq. (5) using the bulk viscosity.

**Figure 9:**
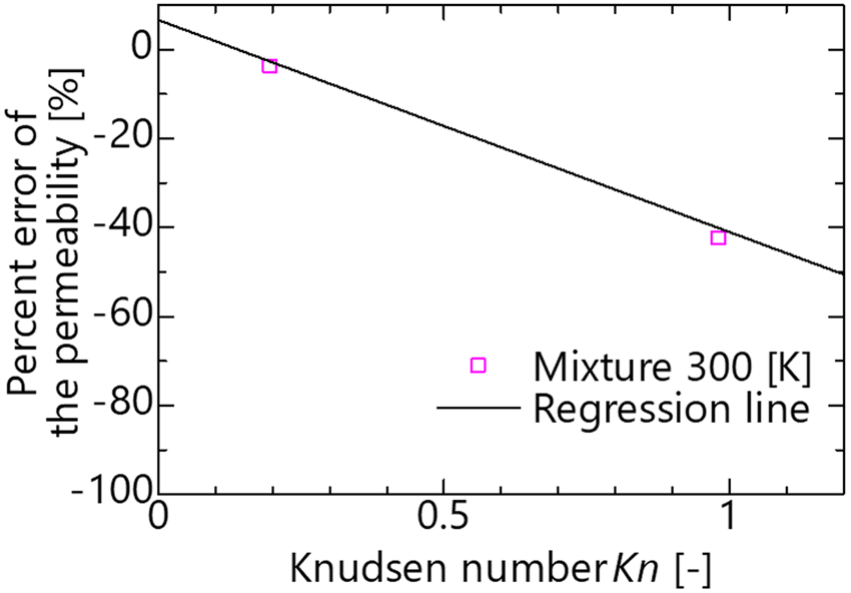
Percent error of the permeability as a function of Knudsen number based on results of aqueous ethanol. The solid line indicates the regression line of Fig. 6.

To demonstrate that these results are also the same in the mixture solution assumed at the time of actual filtration and resin impregnation, we conducted a permeation simulation using an aqueous ethanol solution. An aqueous ethanol solution with a mole fraction of 0.5 at 300 K is used. First, the relaxation is calculated using an NPT ensemble for an aqueous ethanol solution with a total number of molecules of 3,000 (water: ethanol = 1500: 1500 molecules) for 1.0 ns, and the physical property values are then calculated by calculating the NVT ensemble for 0.2 ns. The permeabilities at *d*_0_ = 1.0 and 3.0 are then calculated. If the obtained percent error of permeability is present on the regression line of Fig. 6, it indicates that the applicability of the continuum law can be predicted as a linear equation of the Knudsen number in a mixed liquid. The density obtained from the NPT ensemble simulation is 858.0 kg/m^3^, and the viscosity coefficient is 1.589 mPa∙s. These values obtained from the Jouyban-Acree model in the literature are 852.9 kg/m^3^ and 1.729 mPa∙s, respectively^21^. Because the error from the physical property values obtained are less than 10%, the simulation conditions are appropriate. Next, to show that the percent error of the permeability can be predicted even with a mixed fluid, the percent errors obtained are plotted in pink squares in Fig. 9. Here, 0.491 nm calculated from Eq. (2) is used as the molecular diameter. It was confirmed that the errors between the regression line and the measurement point are within 3% for *d*_0_, and are approximately along the regression line. Therefore, the law of continuum can also be predicted based on the linear equation of the Knudsen number in a mixed liquid. Therefore, in the range of *K*_*n*_ < 1.0, the deviation from the continuum model can be approximated as a linear function of the Knudsen number regardless of the channel width, temperature, and molecular type as follows.

7 $$\frac{{K}_{MD}-{K}_{eq}}{{K}_{eq}}=-\,47.63\,Kn+6.62$$

Since *K*_*MD*_ is assumed to be the actual permeability, the predicted permeability *K* is obtained from above equation and is calculated as follows.

8 $$K=\frac{\pi {d}^{2}}{32\,\mu }(7.62-47.63\,Kn)$$

This equation suggests that the permeability can be calculated only with the channel width *d*, viscosity coefficient *μ*, and the Knudsen number, and it is not necessary to calculate complicated nano physical properties.

## Discussion

In this study, to specify the parameters affecting the magnitude of divergence from the continuum dynamics model in a nanoscale flow, we conducted a fluid permeation simulation using molecular dynamics and determined the permeability by changing the channel width, fluid temperature, and fluid type. The following findings were obtained.

1. The influence of the fluid temperature and type of fluid molecules on the amount of divergence from the continuum model is small compared to that of the channel width.
2. Within the range of *Kn* < 1.0, the amount of divergence from the continuum model can be approximated as a linear function of the Knudsen number regardless of the channel width, temperature, and molecular type.

A high separation performance is expected in the filtration field when the Knudsen number is close to 1, but is outside the application range of the continuum mechanics within the range of 0.1 < *Kn* < 1.0, as shown in Table 1. We have previously reported on the continuum model. However, this model has been found to diverge if the flow width becomes too narrow, and many researchers are working on making a more accurate model, as stated in the Introduction. Their models have yielded excellent predictions, but permeability predictions are considered costly because their models require nanoscale properties that are difficult to measure. On the other hand, our study does not require the data of nanoscale physical properties that are difficult to measure and showed that prediction is possible by utilizing only the bulk physical properties of the fluid and the channel width, which are macro physical properties. In this respect, this study provides a more practical proposal than did previous studies.

## Methods

All simulations were conducted using the Large-scale Atomic Molecular Massively Parallel Simulator^22^, and an OPLS-AA force field^23,24^ was used to describe all atoms with a time step of 1 fs. The temperature in the system was controlled using a Nosé-Hoover chain thermostat^25^. Periodic boundary conditions were applied in all three dimensions. In the OPLS force field, the noncovalent interactions represented by the Van der Waals and electrostatic terms are expressed through the sum of the Lennard-Jones (LJ) and Coulomb potentials. The cut-off distance for the LJ potential was 1.0 nm. The long-range electrostatic interactions at over 1.0 nm were calculated using the particle-particle particle-mesh^26^ method. The Lorentz-Berthelot rule was used for the LJ parameter between heterogeneous atoms. For the water molecules, the TIP4P2005 model^27^ was used; in this model, water is treated as a simple rigid body. The SHAKE algorithm^28^ was applied to the bond length and angle between the oxygen and hydrogen atoms of the water molecules to prevent high-frequency vibrations that shorten the unit time step. Originally, force fields and molecular models were modeled to reproduce macro properties. Our target fluid field is determined by the macro properties of density and viscosity. It is confirmed by the equilibrium simulation described late that these values agree well with the experimental values. Therefore, it is clear that our chosen force field and molecular model are valid.

The physical properties were calculated through an equilibrium simulation using only fluid molecules. In the case of water and ethanol, 1,718 and 3,200 molecules are placed in the cell, respectively. The initial systems were applied in an NPT ensemble at 1 atm for 1 ns and the density was determined. The viscosity coefficient was then calculated using an NVT ensemble for 2.0 ns. The viscosity coefficient was calculated from the Green-Kubo equation^29^.

9 $$\mu =\frac{V}{{k}_{B}T}{\int }_{0}^{\infty }\,{\langle {P}_{\alpha \beta }({t}_{0})\otimes {P}_{\alpha \beta }({t}_{0}+t)\rangle }_{t0}dt$$

where the part inside < > indicates the time average of the autocorrelation function of the pressure tensor *P*_*αβ*_ with direction components *α* and *β*. For the integration interval, it is necessary to have a sufficiently long finite time until the value converges, whereas if the time is long, the number of samples decreases and the error increases^30^. Therefore, to determine the convergence value, the sampling length was changed from 1 to 20 ps every 1 ps, the data obtained were approximated as a logistic regression, and the value at *t* → *∞* was used.

For a fluid permeation simulation, the cell size is *l*_*x*_ = 2.9 nm, *l*_*y*_ ≧ 3*d*_0_, and *l*_*z*_ was determined based on an NPT ensemble calculation, which had 3,200 ethanol molecules randomly arranged in the cell (8,900 at *d*_0_ = 5.0 nm)^31^ at 1 atm for 1 ns while allowing a deformation in only the z direction. The graphene slits were arranged perpendicular to the *z* direction at the position of *z* = *l*_*z*_/2 with slit width *d*. Each carbon atom constituting the graphene slit is fixed in space, and therefore does not flow. For each atom located within a region of 0.5 nm in thickness (red part in Fig. 1(a)) at one end in the z-axis direction of the cell, the constant force is *f*_*z*_ ( = 3.598 × 10^6^). By the same external force addition method, it has been reported that the permeation phenomenon close to the experiment can be reproduced^32^. Flow calculations were conducted for 1 ns (Supplementary Movie 1).

The pressure was calculated using the virial theorem^33^.

10 $$\begin{array}{rcl}\langle P\rangle  & = & \frac{N{k}_{B}\langle T\rangle }{V}+\frac{1}{3{\rm{V}}}\mathop{\sum }\limits_{i=1}^{N}\,\mathop{\sum }\limits_{\begin{array}{c}j=1\\ j\ne i\end{array}}^{N^{\prime} }\,\langle {{\bf{r}}}_{ij}\cdot {{\bf{F}}}_{ij}\rangle \\ {{\bf{r}}}_{ij} & = & {{\bf{r}}}_{i}-{{\bf{r}}}_{j}\end{array}$$

where *V* is the volume, *T* is the temperature, *N* is the number of atoms in the region, *N*′ is the number of atoms interacting with atom *i* including the periodic boundary condition, *k*_*B*_ is the Boltzmann constant, and **F**_*ij*_ is the force that atom *i* receives from atom *j*. The pressures obtained by applying Eq. (10) to the local region before and after the membrane permeation are defined as *P*_*before*_ and *P*_*after*_, and the difference is set as Δ*P*.

11 $${\rm{\Delta }}P={P}_{before}-{P}_{after}$$

The flow rate *Q* was obtained from the number of molecules *dN* permeated during time *dt*.

12 $$Q=\frac{M}{\rho {l}_{x}{N}_{A}}\frac{{\rm{dN}}}{{\rm{dt}}}$$

where *M* is the molecular weight, *N*_*A*_ is an Avogadro constant, *ρ* is the fluid density, and *l*_*x*_ is the unit cell length for the *x* direction. When dividing by *l*_*x*_, the flow rate is converted into the unit length along the *x* axis direction. The error bars attached to each value indicate the standard error of the values sampled every 10 ps during each measurement unless otherwise noted.

## Supplementary information

Supplementary movie 1
